# Effects of ondansetron on respiratory pattern and sensation of experimentally induced dyspnea

**DOI:** 10.1590/S1516-31802002000500004

**Published:** 2002-09-02

**Authors:** José Antônio Baddini Martinez, Fábio Senra Rocha, Elizabet Sobrani, Fabíola Paula Lovreto Galhardo, João Terra

**Keywords:** Dyspnea, Ondansetron, Pulmonary Function, Analog Scale, Dispnéia, Ondansetrona, Função Pulmonar, Escala Analógica

## Abstract

**CONTEXT:**

Dyspnea remains a therapeutic challenge, especially in chronic respiratory conditions. Recent studies have shown that the induction of unpleasant dyspnea sensations activates areas in the insular cortex.

**OBJECTIVE:**

This study was designed to investigate the potential effects of ondansetron, a potent anti-serotonin agent, on induced dyspnea sensation.

**TYPE OF STUDY:**

A randomized double blind study.

**SETTING:**

Pulmonary Function Laboratory of Hospital das Clínicas de Ribeirão Preto.

**PARTICIPANTS:**

Ten healthy male volunteers (mean age ± standard error = 23.1 ± 0.41 years) without respiratory diseases and showing normal spiro-metric tests.

**INTERVENTIONS:**

Uncomfortable breathing was induced in the volunteers on two different days, via the use of inspiratory resistors (loads of 0, 7, 14 and 21 cm H_2_O/l/sec) and breathholding, two hours after taking 8 mg of ondansetron (Ond) or placebo (Plac).

**MAIN MEASUREMENTS:**

Respiratory discomfort during breathing under loading was evaluated on a 100-mm visual analog scale. The maximum length of time of voluntary apnea was measured in seconds.

**RESULTS:**

The mean maximum voluntary apnea time did not differ between the ondansetron and placebo days (Plac = 96 ± 6.6 sec vs. Ond = 100 ± 7.9 sec). Ondansetron did not influence the dyspnea sensation induced by different inspira-tory loads (0 cm H_2_O/l/sec: Ond = 1.4 mm ± 0.44 vs. Plac = 2.1 ± 0.85 mm; 7 cm H_2_O/l/sec: Ond = 16.6 ± 2.74 mm vs. Plac = 13.7 ± 2.06 mm; 14 cm H_2_O/l/sec; Ond = 30.5 ± 4.50 mm vs. Plac = 27.1 ± 3.44 mm; 21 cm H_2_O/l/ sec: Ond = 50.3 ± 6.72 mm vs. Plac = 49.4 ± 6.72 mm). Ondansetron led to significant decreases in tidal volume under basal conditions and when breathing under the highest inspiratory loading (0 cm H_2_O/l/sec: Ond = 0.83 ± 0.26 l vs. Plac = 1.0 ± 0.28 l; 21 cm H_2_O/l/sec: Ond = 0.86 ± 0.23 l vs. Plac = 1.1 ± 0.22 l)

**CONCLUSION:**

The present results suggest that 5-HT3 receptors do not play an important role in the mediation of dyspnea sensations.

## RESUMO

**CONTEXTO:**

O tratamento da dispnéia continua sendo um desafio terapêutico, principal-mente nos casos de insuficiência respiratória crônica. Estudos recentes mostraram que a indução de sensações respiratórias desagradáveis ativa áreas do córtex insular.

**OBJETIVO:**

Este estudo foi concebido para investigar os efeitos potenciais da ondansetrona, um agente anti-serotononinérgico potente nas sensações de dispnéia induzida.

**TIPO DE ESTUDO:**

Estudo duplo-cego randomizado.

**LOCAL:**

Laboratório de Função Pulmonar do Hospital das Clínicas de Ribeirão Preto.

**PARTICIPANTES:**

10 homens sadios (idade média ± EPM = 23,1 ± 0,41 anos) sem doenças respiratórias e mostrando testes espirométricos normais.

**INTERVENÇÃO:**

Os voluntários desenvolveram respiração desconfortável em duas diferentes ocasiões, através do uso de resistências inspiratórias (cargas de 0, 7, 14 e 21 cm H_2_O/l/sec) e apnéia voluntária, duas horas após tomarem 8 mg de ondansetrona (Ond) ou placebo (Plac).

**VARIÁVEIS ESTUDADAS:**

O desconforto respiratório durante a respiração com cargas foi avaliado por uma escala analógica visual de 100 mm. O tempo máximo de apnéia voluntária foi medido em segundos.

**RESULTADOS:**

O tempo máximo de apnéia médio não diferiu entre os dias em que foi administrado ondasetrona ou placebo (Plac = 96 ± 6,6 s X Ond = 100 ± 7,9 s) e a droga não influenciou a sensação de dispnéia induzida por diferentes cargas inspiratórias (0 cm H_2_O/l/sec: Ond = 1,4 mm ± 0,44 X Plac = 2,1 ± 0,85 mm; 7 cm H_2_O/l/sec: Ond = 16,6 ± 2,74 mm X Plac = 13,7 ± 2,06 mm; 14 cm H_2_O/l/sec; Ond = 30,5 ± 4,50 mm X Plac = 27,1 ± 3,44 mm; 21 cm H_2_O/l/sec: Ond = 50,3 ± 6,72 mm X Plac = 49,4 ± 6,72 mm). Ondansetrona levou a decréscimos significantes do volume corrente em condições basais e quando respirando com a maior carga inspiratória (0 cm H_2_O/l/sec: Ond = 0,83 ± 0,26 l X Plac = 1,0 ± 0,28 l; 21 cm H_2_O/l/sec: Ond = 0,86 ± 0,23 l X Plac = 1,1 ± 0,22 l)

**CONCLUSÃO:**

Os resultados atuais sugerem que receptores serotononinérgicos do tipo 5-HT3 não desempenham um papel importante na mediação das sensações de dispnéia.

## INTRODUCTION

Dyspnea is one of the most common complaints among patients with cardiorespiratory diseases and is often the reason why a person seeks medical attention.^1,2^ The presence of unpleasant respiratory sensations is the most important symptom affecting quality of life in patients with chronic respiratory failure.^3,4^ Al-though our knowledge about the physiological mechanisms that generate the respiratory sensations has increased over the past years, dyspnea still remains a formidable clinical problem and a therapeutic challenge. An agent capable of reducing dyspnea, without significant side effects, would never be a substitute for specific therapy aimed at the underlying respiratory disorder, but could be useful, especially when the condition is irreversible.^5^

Knowledge of dyspnea pathogenesis necessarily involves the understanding of nervous mechanisms involved in the generation, transmission and modulation of respiratory rhythm, both at the peripheral and central nervous system (CNS) levels.^1,2,6^ The most important neurotransmitters involved in these phenomena at CNS level appear to be glutamate, gamma-aminobutyric acid (GABA), glycine and serotonin (5-hydroxytryptamine, 5-HT). Indeed, it has been shown that 5-HT has diverse and complex effects on respiratory physiology.^7^

Ondansetron is a 5-HT3 receptor antagonist used for preventing and treating vagally mediated emesis caused by anticancer chemo-therapy and in the immediate postoperative period.^8^ Substantial experience of this drug has been gained over the past decade, together with increased assurance through its use.^9^ In addition, new uses for ondansetron have been described, including the reduction of drinking among subjects predisposed to alcoholism, anxiolysis, attenuation of age-associated memory impairment, anti-psychotic effects in Parkinson's disease, and improvements in migraine and nervous bulimia patients.^10–14^ Ondansetron also promotes reduction of digestive secretions and diarrhea caused by increased intestinal serotonin content and a decrease in experimentally induced pain.^14,15^ Even though a substantial number of pharmacological actions have been demonstrated for ondansetron, no study has yet been published dealing with its potential effects on breathlessness.

The present study was designed to investigate the effects of oral ondansetron versus placebo on respiratory sensations in a group of healthy volunteers in whom uncomfortable breathing was induced via the use of inspira-tory resistors and maintaining voluntary apnea.

## METHODS

### Subjects

Ten non-smoking, healthy male volunteers participated in the study after giving informed consent. None of the subjects had respiratory symptoms or past pulmonary illnesses and all had a normal physical examination. Special care was taken to exclude subjects with a history of asthma or wheezing during childhood. The ages ranged from 21 to 26 years, with a mean ± standard error of 23.1 ± 0.4 years. The study was approved by the Ethics Committee of Hospital das Clínicas de Ribeirão Preto.

### Study design

Dyspneic sensations were induced by breathholding and breathing under different inspiratory resistive loadings. The volunteers came to the pulmonary function laboratory three times. On the first day (D-1), they performed spirometry and dyspnea tests in order to familiarize themselves with the maneuvers. On the second (D-2) and third days (D-3) they were medicated with placebo or with 8mg of per oral ondansetron two hours before the tests. Drug or placebo was administered at random and in a double-blind manner. All tests were performed around midday at least two days apart, and with the subjects breathing room air.

### Spirometry

Basal pulmonary function data were obtained using a Pulmonet Godard spirometer. Procedures were performed according to the American Thoracic Society recommendations.^16^ Forced vital capacity (FVC), forced expiratory volume in the first second (FEV_1_) and the middle curve forced expiratory flow (FEF_25-75%_) were analyzed in comparison with predicted normal values. The FEV_1_/FVC ratios were expressed as percentage values. The predicted normal values were based on Crapo et al.^17^

### Breathholding

When the volunteers arrived at the pulmonary function laboratory they received an explanation of the proceedings and, after a brief resting period, were asked to hold their breath at the total lung capacity level, the most they could. An observer employed a chronometer to measure the length of apnea, while the volunteers used hand signaling to indicate the beginning and end of the breathholding period. The maximum voluntary apnea time was measured twice, at a separation of at least 15 minutes, and the duration was expressed in seconds. The best value obtained between the two maneuvers was chosen for analysis.

### Breathing with inspiratory loads

Each subject breathed through an oral one-way valve system during the experiment. A nose clip was placed to assure no air leaks. The valve expiratory port was connected to a Collins Multimodular Lung Analyzer (model 02303) in order to register the respiratory pattern and tidal volume using a graphic ink system. End-tidal carbon dioxide tension (PETCO_2_) was measured using a CO_2_ analyzer via a port in the expiratory limb. The same equipment was fitted with a pulse oximeter that was connected to one finger so as to continuously monitor the arterial oxygen saturation (DX 7100 ETCO_2_/SPO_2_ reader, Dixtal, SP). An adjustable resistor device employed in respiratory muscle training (Threshold IMT^Ò^, Healthscan, NJ) was connected to the valve inspiratory limb in order to introduce variable respiratory loads. The volunteers were asked to breath into the system without the resistor device (zero load) and with loads of 7, 14 and 21 cm H_2_O/l/sec. The different loads were applied at random and in a blinded manner for the volunteers. The subjects breathed for a period of two minutes at each load and, at the end of the second minute, their respiratory data were recorded and they were asked to rate the degree of respiratory discomfort using a visual analog scale (VAS).^18^ The analog scale consisted of a vertical 100-mm line with the number zero at the bottom and the number 100 at the top. The volunteers were instructed to consider zero as the indication of no respiratory sensation at all and 100 as a sensation that was intolerable.

### Statistical Analysis

Data are reported as means plus standard errors. Comparisons of the measurements obtained with ondansetron and placebo were made using the paired-t test or Wilcoxon non-parametric test. A value of p < 0.05 was considered to be significant.

## RESULTS

All the volunteers showed normal spiro-metric parameters. The mean values and standard errors for the pulmonary function data were: FVC = 95 ± 2.1 %, FEV_1_ = 94 ± 1.7 %, FVC/FEV_1_ = 98 ± 1.9 %, and FEF_25-75%_ = 92 ± 2.3 %. Five volunteers received placebo on D-2 and ondansetron on D-3, and the remaining five subjects took the drugs in an inverse order. The mean time interval between D-2 and D-3 was 4.1 ± 0.4 days (range: 2 to 6 days).

**Figure 1:**
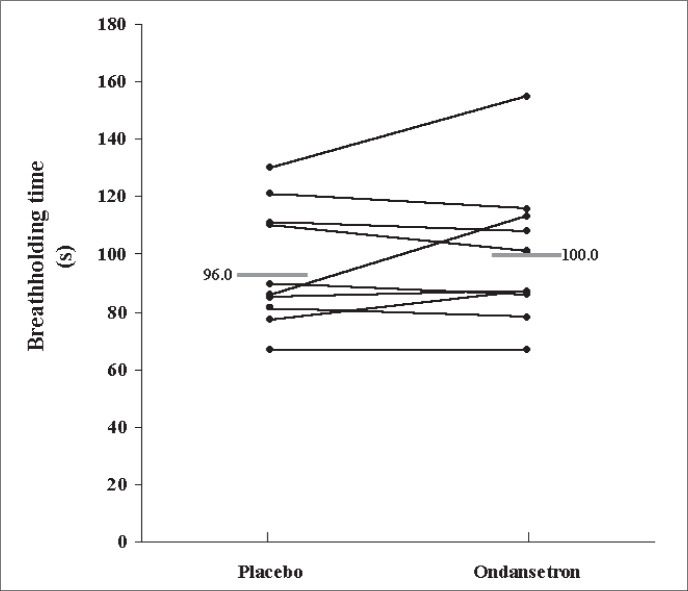
Maximum voluntary length of apnea for 10 volunteers after taking placebo or 8 mg of ondansetron.

The mean maximum voluntary apnea time did not differ between the ondansetron and placebo days (placebo = 96 ± 6.6 s vs. ondansetron = 100 ± 7.9 s; Figure 1).

Ondansetron did not lead to changes in respiratory rate or arterial oxygen saturation at any load, in comparison with placebo (Table 1). The drug intake was associated with significant decreases in tidal volume under basal conditions and with respiration under an inspiratory load of 21 cm H_2_O/l/sec. However, there were no differences between drug and placebo days with regard to minute ventilation for any respiratory load. A significant increase in PETCO_2_ was also observed during respiration using an inspiratory load of 21 cm H_2_O/l/sec after ondansetron administration.

**Table 1:** Respiratory parameters measured at different inspiratory loads

Respiratory	Placebo or	Inspiratory load			
Parameter	Ondansetron	0 cm H_2_O/l/s	7 cm H_2_O/l/s	14 cm H_2_O/l/s	21 cm H_2_O/l/s
RR (rpm)	Placebo	13.0 ±1.77	14.0 ±2.37	12.9 ±1.99	12.8 ±1.81
	Ondansetron	13.6 ±1.48	14.5 ±1.58	14.5 ±1.86	13.4 ±1.77
TV(l)	Placebo	1.0 ±0.28 *	1.1 ±0.25	1.1±0.28	1.1 ±0.22 *
	Ondansetron	0.83 ±0.26	0.90 ±0.18	0.98 ±0.26	0.86 ±0.23
VE(l)	Placebo	10.6 ±1.34	11.0 ±1.36	10.8 ±1.44	9.9 ±0.88
	Ondansetron	9.04 ±1.33	10.4 ±0.97	10.1 ±1.26	9.39 ±1.09
PETCO_2_ (mmHg)	Placebo	35.3 ±1.75	33.8 ±1.97	34.0 ±2.15	33.8 ±2.04 *
	Ondansetron	37.5 ±1.92	36.2±2.06	36.1 ±2.18	37.3 ±2.13
SaO_2_ (%)	Placebo	97.5 ±0.17	97.5±0.27	97.6 ±0.27	97.6±0.27
	Ondansetron	97.4 ±0.22	97.2 ±0.29	97.1 ±0.28	97.5 ±0.27

*RR: respiratory rate; rpm = respirations per minute; TV: tidal volume; VE: minute ventilation; SaO_2_ = PETCO_2_ : end tidal expiratory CO_2_,*

* *p< 0.05 by paired-t test comparing placebo vs. ondansetron.*

**Figure 2:**
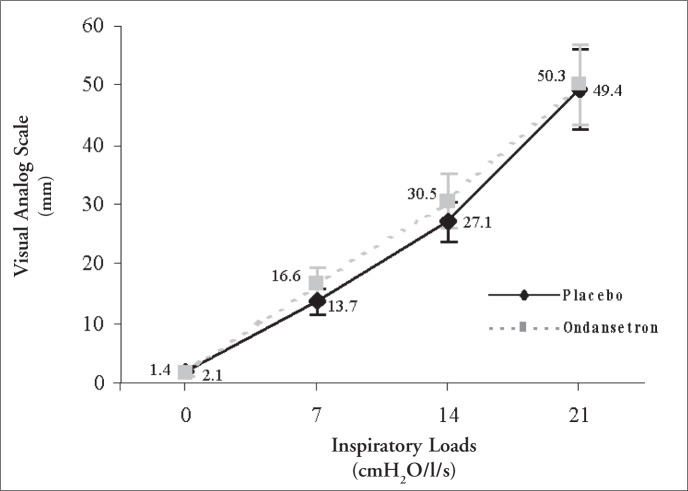
Mean dyspnea sensations according to inspiratory loads and type of treatment.

The use of ondansetron did not influence the mean dyspnea sensation induced by different inspiratory loads (Figure 2).

## DISCUSSION

Dyspnea may be defined as an uncomfortable awareness of difficult breathing or the need to breath. Dyspnea, like pain, is a very common symptom in several diseases. In addition, dyspnea is a multidimensional sensory experience involving affective components and complex nerve processing.^1,2^ Numerous peripheral afferent pathways are involved in the development of breathlessness, including chemoreceptors and mechanoreceptors located in the upper airways, lungs and chest wall.^1,2,6^ The present evidence indicates that dyspnea results from multiple and simultaneous sensory inputs, emphasizing a fundamental role for the CNS in the final integration of this complex sensation. However, until recently little was known about the brain regions involved in these processes.

New investigations with the use of positron emission tomography have shown activation of specific areas of the brain in response to different dyspnea-inducing stimuli. Banzett et al.,^19^ employing a non-invasive system, ventilated normal volunteers with low tidal volumes, while keeping arterial oxygen and carbon dioxide levels constant. They observed an increase in regional cerebral blood flow (rCBF) at the right insular cortex. Peiffer et al.^20^ induced respiratory discomfort in healthy volunteers by adding external resistive loadings during inspiration and expiration. Neural activation was shown in three distinct brain regions: the right anterior insula, the cerebellar vermis and the medial pons. Thus, two different research groups using distinct stimuli to generate respiratory discomfort found an association between dyspnea and activation of the insular cortex. The insula is a limbic structure activated by different visceral stimuli, including temperature, taste, pain and nausea.^19,21–23^ Like dyspnea, such perceptions underlie behaviors essential for homeostasis and survival. Vagal afferents, including respiratory chemoreceptors and pulmonary receptors, project to the posterior insula.^24^

Different authors have explored various drugs as a means for alleviating dyspnea in chronic respiratory conditions. It is presumed that such medications could act by altering perceptual sensitivity or exerting respiratory depressive effects in the CNS.^1,5^ Examples of this type of agents are opiates and anxiolytics. In addition, there are also suggestions that oral indomethacin and inhaled furosemide may positively interfere with the mechanisms that give rise to breathlessness.^25,26^

In the present study we investigated a possible role for ondansetron as an agent capable of reducing unpleasant respiratory sensations elicited by two distinct mechanisms: breath-holding and inspiratory loadings. This type of investigation had not previously been done. Although the current clinical use of ondansetron is exclusively as an anti-emesis agent, a great number of additional uses for the drug have been described.^9–14^ Moreover, there is evidence to support a role for 5-HT receptors in neural respiratory control.^7^ Serotonin may be particularly important in metabolic states such as hypoxia or ischemia that cause apneustic breathing, since 5-HT restores normal breathing pattern under these conditions.^7^

Systemic administration of large doses of 5-HT agonists has been shown to decrease the discharge frequency of inspiratory and expiratory neurons. Smaller doses produce the opposite effect, increasing the discharge frequency and decreasing the burst duration of inspiratory units and causing expiratory neurons to fire earlier in relation to phrenic nerve activity.^7^ Serotonin is also a potent bronchoconstrictor and pulmonary vasoconstrictor.^27^ The variability of the respiratory effects of 5-HT may be, in part, due to the subtypes of receptors preferentially expressed at the periphery and in CNS neurons. On the basis of responses to relatively selective serotonin receptor antagonists and agonists, seven classes of 5-HT receptors have been characterized.^15^

Ondansetron is a specific 5-HT3 receptor antagonist with negligible agonistic or antagonistic activity at alpha or beta-adrenergic and histamine, muscarinic, central or peripheral dopaminergic receptors.^14,15^

Our results show that the oral administration of 8 mg of ondansetron two hours before the studies did not significantly improve the breathholding time or the degree of respiratory discomfort induced by different inspira-tory loads in a group of 10 healthy young volunteers, but it led to small changes in respiratory physiological parameters (Figures 1 and 2 and Table 1). Decreases in tidal volume associated with drug intake were observed at all respiratory loads, although they reached statistical significance only under basal conditions and when breathing under a load of 21 cm H_2_O/l/sec. There was also a trend towards increased respiratory rate and decreased minute ventilation, but without statistical significance. Following the trend of reductions in minute ventilation, PETCO_2_ increased with ondansetron administration, but it only reached a statistical difference in comparison with placebo at an inspiratory load of 21 cm H_2_O/l/sec.

It is recognized that 5-HT3 receptors are present on vagal afferent fibers in the gastrointestinal tract and probably in the respiratory system as well.^15,28^ The brain regions with a high density of 5-HT3 receptors are the area postrema, olfactory bulb and amygdala.^29^ Ondansetron is a drug that is well absorbed from the gastrointestinal tract and rapidly penetrates the brain barriers.^8^ Peak concentrations in the bloodstream following oral administration are usually achieved within 0.8 to 2 hours. Oral doses of 8 mg have shown anti-emetic action in patients submitted to highly emetogenic chemotherapy.^14,15^ The current results therefore most probably represent the overall respiratory consequence of 5-HT3 receptor blocking both at the peripheral and CNS levels.

Animal studies have shown that the administration of high doses of ondansetron significantly increases the rCBF in the raphe nuclei.^30^ Lower doses reduced rCBF in 13 of 66 brain regions studied, including visual and auditory systems, the lateral habenula and, more markedly, the limbic system. The latter metabolic effects are common to other agents with anxiolytic activity, like diazepam, and are possibly related to the higher 5-HT3 receptor densities in the hippocampus.^31^ These studies clearly demonstrate that the overall rCBF effect of ondansetron does not necessary correspond to the known distribution of 5-HT3 receptors. Such a discrepancy may be explained by the fact that the drug may indirectly modulate multiple neurotransmitter release by ligation to 5 HT3 receptors containing neurons.^31^

Dyspnea is a unique experience, for which the only reliable measurement is the patient's own report. Pulmonary function parameters, like respiratory rate, arterial blood gases and spirometric values, do not show strong correlation with breathlessness, nor can they explain all the intensity of feelings.^32^ There are no animal models for evaluating breathlessness. Therefore, studies of induced dyspnea in healthy volunteers are valid tools for investigating its physiopatho-logical mechanisms and the potential therapeutic value of new medicines. In this study, ondansetron did not interfere with the dyspnea sensation induced by breathholding and inspiratory loading. Such findings suggest that 5-HT3 receptors are most probably not related to the development of unpleasant respiratory awareness. However, it is worth emphasizing that this study did not evaluate the chronic effects of ondansetron on breathlessness. The administration of the drug for longer periods of time might have yielded different results.

## CONCLUSION

The oral administration of 8 mg of ondansetron to healthy volunteers led only to minimal alterations in respiratory patterns and no changes in the degree of dyspnea induced by voluntary apnea and breathing under inspiratory loads. These results suggest no role for 5-HT3 receptors in the genesis of the breathlessness sensation.
